# Distribution of health problems at the general outpatients' clinic of the University of Hong Kong-Shenzhen Hospital: A cross-sectional study

**DOI:** 10.3389/fpubh.2022.889819

**Published:** 2022-08-08

**Authors:** Kai Chen, Zhuo Li, Ruihong Liu, Yanyan Zhu, Weihui Yan, Ng Amy Pui Pui, Zhiyuan Chen

**Affiliations:** ^1^Department of General Practice, Shenzhen Hospital, The University of Hong Kong, Shenzhen, China; ^2^Li Ka Shing Faculty of Medicine, The University of Hong Kong, Shenzhen, China

**Keywords:** general practice, health problem, distribution, primary care, chronic disease

## Abstract

**Objective:**

The study aimed to understand the distribution of health problems of a general practice clinic to provide guidance on how to develop primary care in Shenzhen, China.

**Study design:**

This is a cross-sectional study.

**Methods:**

Patients' sociodemographic data and diagnoses were recorded from the electronic medical record system of the University of Hong Kong-Shenzhen Hospital from Jan 2014 to Dec 2020 and coded using the International Classification of Primary Care-2. Descriptive statistics were used to describe the distribution of health problems.

**Results:**

A total of 368,167 health problems were recorded. Respiratory, digestive, musculoskeletal, general, and cardiovascular were the top five categories, which accounted for 67.71% of the total in this study. Acute upper respiratory tract infection (AURTI) was the most common health problem (6.67%). Chronic diseases, including hypertension and diabetes mellitus, accounted for about 7% of all health problems. The proportion of cardiovascular problems increased significantly with age (r = 0.96, *P* < 0.05). The proportion of consultations for mental health problems was low in all age groups.

**Conclusions:**

The results represent an understanding of the common health problems of patients in Shenzhen city, which can provide a reference for preventing diseases and developing primary care.

## Introduction

Primary care has received considerable attention since it was introduced into China in the 1980s and has been a significant issue in China's healthcare reform (1). To strengthen the quality of primary health services, the Chinese government has implemented several policies, including financial support, personnel training, pharmaceutical systems, and medical insurance (2, 3).

Shenzhen, a special economic zone in southern China neighboring Hong Kong, is world-renowned for its rapid-growing economic and innovative technology. Shenzhen has a large immigrant population from other parts of China who come to Shenzhen temporarily for work purposes, and, as a result, the population is skewed toward younger adults (4). Shenzhen began to develop primary health services in 1996 and widely implemented a gatekeeping system as a core policy of primary care (5). In Shenzhen, patients access primary care in community health centers and in the general outpatients' clinic of the general hospital. Apart from treating illness, primary care physicians are involved in health promotion and disease prevention. There are over 700 community health centers in Shenzhen, which provides 33.5 million clinical visits per year and accounts for over 30% of clinical visits in the entire medical system. Due to the success of medical reform, the residents' average life expectancy in Shenzhen is 81 years, close to that in the UK (6).

To further optimize primary health services and allocate medical resources, it is necessary to understand patients' health problems in primary care. Prevalence of health problems can reflect the community's general health and show trends, providing valuable indicators on health care needs and health services planning (7). Based on international experience, the International Classification of Primary Care-second edition (ICPC-2) was chosen to describe health problems in primary care (8, 9). ICPC-2 was developed by the WONCA International Classification Committee, including classifications of complaints and diagnoses, and is a patient-oriented rather than disease-or provider-oriented approach (10). Several studies have used ICPC-2 to assess health problems in China, but few focused on those in Shenzhen (11, 12).

The University of Hong Kong-Shenzhen Hospital is a Tri-Service General Hospital invested by the Shenzhen municipal government, which introduced the hospital management mode and advanced medical technology of the University of Hong Kong. The hospital adheres to the concept of using general practice as a gatekeeper to the specialties. Most health problems can be solved at the general outpatients' clinic with the trained general practitioner and comprehensive inspection means. This mode may assess more health problems than in community health centers (13).

This study aimed to investigate health problems at the general outpatients' clinic of the University of Hong Kong-Shen Zhen Hospital to provide evidence for developing policies on health services planning and general practitioners (GPs) training in Shenzhen city.

## Methods

### Study design

A cross-sectional study was conducted at the University of Hong Kong-Shenzhen Hospital's general outpatient clinic from Jan 2014 to Dec 2020. Patients' health problems, including chief complaints or diagnoses, were downloaded from the electronic medical record systems of the University of Hong Kong-Shenzhen Hospital and recoded using the International Classification of Primary Care (ICPC-2).

### Data analysis

Data were recorded into excel spreadsheets with fields for sociodemographic data such as age, sex, date of visit, health problems (including chief complaints or first diagnoses) and ICPC-2 elements of a patient. The data was analyzed using Statistical Product and Service Solutions Version 21 (SPSS 21), and the frequency of ICPC-2 was expressed in terms of percentage distribution.

The Pearson correlation was applied using R (version 3.2.1). A 95% confidence interval accompanied all estimates. A *P*-value of < 0.05 was considered statistically significant.

## Results

### Characteristics of the patients

Characteristics of the patients is presented in Table 1. A total of 368,167 patients and the same number of health problems were recorded in the form of ICPC-2. In all participants, males accounted for 43.64%, and the ratio of males to females was 1:1.29. The average age of the patients was 41 years 6 months [standard deviation (SD) = 14.83], and there was a significant difference between males and females (*P* < 0.05).

**Table 1:** Demographic characteristics of participating parents.

**Age groups**	**Male**	**Female**	**Total**
Pediatric (0–9)	204	131	335 (0.09%)
Adolescent (10–19)	6,045	6,038	12,083 (3.28%)
Young adult (20–44)	100,502	120,477	220,979 (60.02%)
Adult (45–64)	40,711	63,924	104,635 (28.42%)
Elderly (≥65)	13,219	16,916	30,135 (8.19%)
Total	160,681 (43.64%)	207,486 (56.36%)	368,167 (100.00%)
Average age ($\bar{X}\pm$ SD)	40.97 ± 14.56	41.85 ± 15.02	41.47 ± 14.83

Among age groups, young adults (ages 20 to 44) accounted for the most significant proportion (60.02%) of all patients, followed by adults (28.42%), elderly (8.19%), and adolescents (3.28%). In comparison, only a small percentage (0.09%) of the pediatric group seek medical advice at the general outpatients' clinic. Detailed information on the characteristics of the patients is presented in Table 1.

### Distribution of body systems

**Figure 1:**
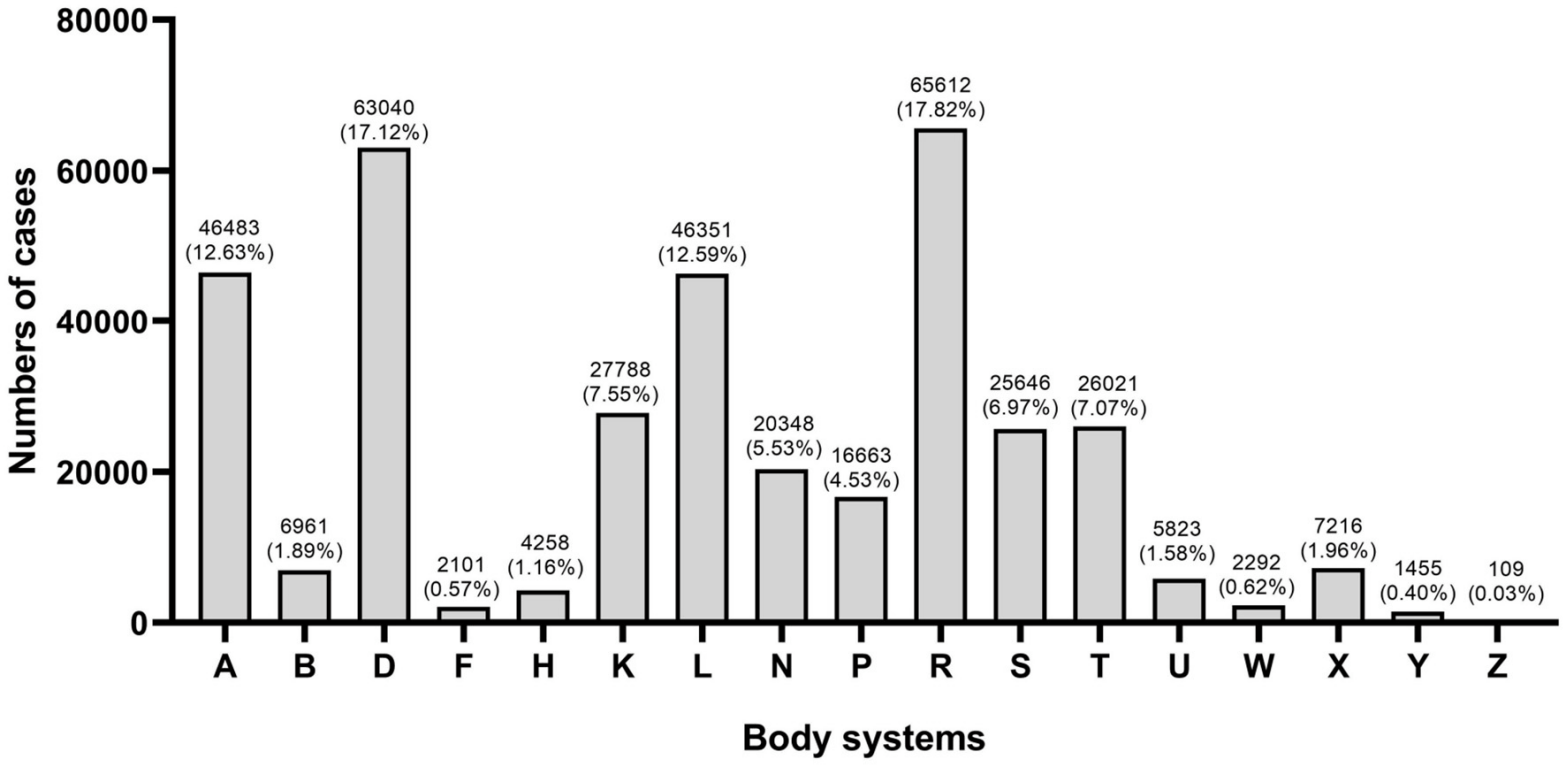
Distribution of health problems by body systems.

The distribution of health problems based on 17 body systems in ICPC-2 is shown in Figure 1. “Respiratory (R),” “Digestive (D),” “Musculoskeletal (L),” “General (A),” and “Cardiovascular (K)” were the top five categories in total, which accounted for 67.71% of the total in this study.

Overall morbidity patterns by age group are shown in Table 2. Respiratory, digestive, musculoskeletal and general problems were common in all age groups. Mainly, the respiratory was one of the most common body systems, which ranked first in all health problems below 45 years. It only ranked fifth (11.38%) in the elderly group, following cardiovascular (21.42%), digestive (12.53%), musculoskeletal (11.92%), and endocrine problems (11.45%). Besides, the proportion of cardiovascular problems had a clear trend that increased significantly with age (r = 0.96, *P* < 0.05).

**Table 2:** Distribution of health problems by age group.

**Body system age group**	**Pediatric (0–9)**	**Adolescent (10–19)**	**Young adult (20–44)**	**Adult (45–64)**	**Elderly (≥65)**
General (A)	50 (14.93%)	1,625 (13.45%)	29,077 (13.16%)	12,608 (12.5%)	3,123 (10.36%)
Blood (B)	7 (2.09%)	337 (2.79%)	4,891 (2.21%)	1,427 (1.36%)	299 (0.99%)
Digestive (D)	37 (11.04%)	2,263 (18.73%)	40,441 (18.30%)	16,524 (15.79%)	3,775 (12.53%)
Eye (F)	9 (2.69%)	70 (0.58%)	1,289 (0.58%)	595 (0.57%)	138 (0.46%)
Ear (H)	0 (0.00%)	77 (0.64%)	2,383 (1.08%)	1,426 (1.36%)	372 (1.23%)
Cardiovascular (K)	0 (0.00%)	277 (2.29%)	8,804 (3.98%)	12,253 (11.71%)	6,454 (21.42%)
Musculoskeletal (L)	11 (3.28%)	974 (8.06%)	26,741 (12.10%)	15,034 (14.37%)	3,591 (11.92%)
Neurological (N)	6 (1.79%)	752 (6.22%)	10,059 (4.55%)	7,318 (6.99%)	2,213 (7.34%)
Psychological (P)	9 (2.69%)	826 (6.84%)	9,465 (4.28%)	5,219 (4.99%)	1,144 (3.80%)
Respiratory (R)	142 (42.39%)	2,870 (23.75%)	46,971 (21.26%)	12,201 (11.66%)	3,428 (11.38%)
Skin (S)	52 (15.52)	1,277 (10.57%)	17,647 (7.99%)	5,274 (5.04%)	1,396 (4.63%)
Endocrine (T)	4 (1.19%)	346 (2.86%)	11,054 (5.00%)	11,167 (10.67%)	3,450 (11.45%)
Urological (U)	3 (0.90%)	122 (1.01%)	3,592 (1.63%)	1,644 (1.57%)	462 (1.53%)
Reproductive (W)	0 (0.00%)	17 (0.14%)	2„174 (0.98%)	95 (0.09%)	6 (0.02%)
Female genital (X)	4 (1.19%)	181 (1.50%)	5,441 (2.46%)	1,530 (1.46%)	60 (0.20%)
Male genital (Y)	1 (0.30%)	61 (0.50%)	872 (0.39%)	299 (0.29%)	222 (0.74%)
Social problems (Z)	0 (0.00%)	8 (0.07%)	78 (0.04%)	21 (0.02%)	2 (0.01%)
Total	335 (100%)	12,083 (100%)	220,979 (100%)	104,635 (100%)	30,135 (100%)

### Distribution of health problems

Table 3 present the top 30 health problems recorded, which accounted for 53.66% of all health problems. Acute upper respiratory tract infection (6.67%) were the most common health problems, followed by abnormal inspection results NOS (3.38%), hypertension without complications (3.29%), cough (3.13%), and vertigo/dizziness (2.81%). Chronic diseases, including hypertension and diabetes mellitus, accounted for about 7% of all health problems.

**Table 3:** Top 30 health problems.

**Rank**	**ICPC-2**	**Complaint/diagnosis**	**Frequency**	**Percent**
1	R74	Acute upper respiratory tract infection (AURTI)	24,547	6.67%
2	A91	Abnormal inspection results NOS	12,459	3.38%
3	K86	Hypertension without complications	12,120	3.29%
4	R05	Cough	11,521	3.13%
5	N17	Vertigo/dizziness	10,353	2.81%
6	D06	Other localized abdominal pain	9,610	2.61%
7	D87	Gastric functional disorder	9,518	2.59%
8	R21	Throat symptom/complaint	7,859	2.13%
9	D70	Gastrointestinal infection	6,458	1.75%
10	T90	Type 2 diabetes mellitus	6,372	1.73%
11	L04	Chest symptoms/complaint	5,647	1.53%
12	R97	Allergic rhinitis	5,600	1.52%
13	T93	Dyslipidemia	5,577	1.51%
14	P06	Sleep apnea	5,327	1.45%
15	A98	Health maintenance/preventive medicine	5,193	1.41%
16	A31	Physical examination/health assessment	5,039	1.37%
17	D02	Abdominal pain	4,796	1.30%
18	A11	Chest pain NOS	4,737	1.29%
19	L18	Muscle pain	4,683	1.27%
20	L03	Lumbar symptom/complaint	4,481	1.22%
21	K02	Chest tightness	4,058	1.10%
22	S88	Allergic dermatitis	3,946	1.07%
23	D72	Viral hepatitis	3,850	1.05%
24	L84	Low back syndrome without radicular pain	3,732	1.01%
25	N01	Headache	3,711	1.01%
26	P74	Anxiety	3,496	0.95%
27	T85	Hyperthyroidism/thyrotoxicosis	3,455	0.94%
28	K87	Hypertension with complications	3,358	0.91%
29	A04	General weakness/fatigue	3,202	0.87%
30	R77	Acute laryngitis/tracheitis	2,920	0.79%

The top 10 health problems by age groups were presented in Table 4. In the pediatric group, allergic rhinitis, AURTI (R74), allergic dermatitis (S88), and cough (R05) accounted for about 40% of all health problems. In adolescent and young adult groups, respiratory problems were the most common, including AURTI, allergic rhinitis and cough. In adult and elderly groups, chronic diseases were the most common, among which hypertension (K86, K87) and diabetes (T89, T90) together accounted for over 20% of all health problems in the elderly group.

**Table 4:** Top 10 health problems in each age group.

	**Pediatric (0–9)**	**Adolescent (10–19)**	**Young adult (20–44)**	**Adult (45–64)**	**Elderly (≥65)**
% of health problems in each age group	Allergic rhinitis (14.63%)	AURTI (9.98%)	AURTI (8.66%)	Hypertension without complications (6.14%)	Hypertension without complications (9.98%)
	AURTI (11.94%)	Allergic rhinitis (4.28%)	Abnormal inspection results NOS (3.78%)	Vertigo/dizziness (3.68%)	Type 2 diabetes mellitus (5.76%)
	Allergic dermatitis (7.16%)	Gastric functional disorder (3.37%)	Cough (3.49%)	Type 2 diabetes mellitus (3.33%)	Hypertension with complications (4.81%)
	Cough (4.78%)	Vertigo/dizziness (2.74%)	Other localized abdominal pain (2.88%)	AURTI (3.25%)	Vertigo/dizziness (4.12%)
	Physical examination (3.58%)	Abnormal inspection results NOS (2.70%)	Throat symptom/complaint (2.68%)	Abnormal inspection results NOS (3.02%)	Cough (2.55%)
	Abnormal inspection results NOS (2.69%)	Cough (2.46%)	Gastric functional disorder (2.55%)	Dyslipidemia (2.94%)	AURTI (2.49%)
	Acute tonsillitis (2.69%)	Other localized abdominal pain (2.23%)	Vertigo/dizziness (2.23%)	Hypertension with complications (2.74%)	Abnormal inspection results NOS (2.05%)
	Mouth/tongue/lip disease (2.09%)	Physical examination (2.22%)	Gastrointestinal infection (2.05%)	Cough (2.62%)	Dyslipidemia (2.05%)
	Fever (1.79%)	Acne (1.75%)	Allergic rhinitis (1.83%)	Other localized abdominal pain (2.33%)	Gastric functional disorder (1.97%)
	Acute bronchitis (1.79%)	Headache (1.70%)	Chest symptoms/complaint (1.67%)	Sleep apnea (2.02%)	Other localized abdominal pain (1.77%)

**Figure 2:**
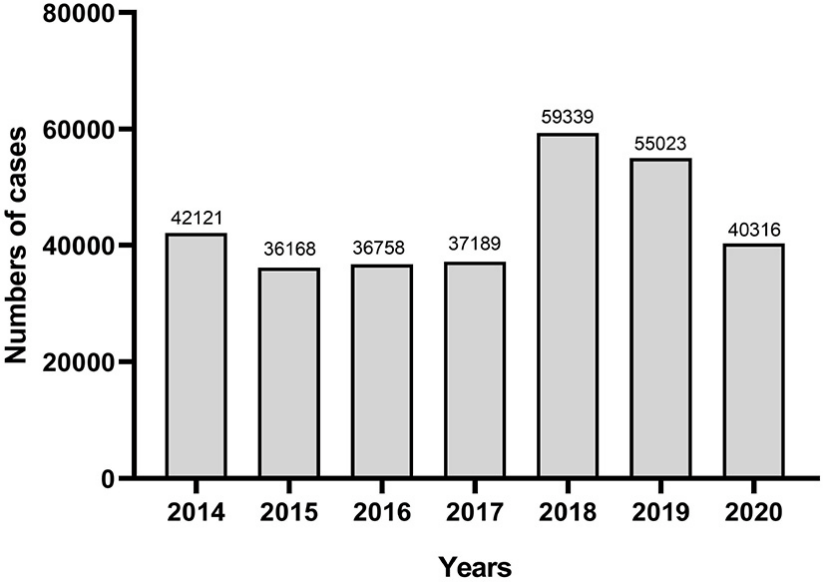
Distribution of cases by years.

Figure 2 and Table 5 shows the distribution of cases by years. In terms of changing trends of case numbers, it shows a rising trend from 2014 to 2019, but a rapid decline in 2020. Acute upper respiratory infection (AURTI) (R74) was the most common problem from 2015 to 2019. However, hypertension without complications (K86) was more frequent in 2020, representing up to 6.05% of the cases.

**Table 5:** Top 10 health problems in in different years.

	**2014**	**2015**	**2016**	**2017**	**2018**	**2019**	**2020**
% of health problems in each age group	Cough (4.54%)	AURTI (5.93%)	AURTI (7.96%)	AURTI (9.62%)	AURTI (8.32%)	AURTI (11.56%)	Hypertension without complications (6.05%)
	AURTI (3.86%)	Other localized abdominal pain (4.07%)	Other localized abdominal pain (3.77%)	Vertigo/dizziness (3.57%)	Abnormal inspection results NOS (4.28%)	Abnormal inspection results NOS (4.51%)	Abnormal inspection results NOS (4.33%)
	Throat symptom/complaint (3.78%)	Cough (3.82%)	Cough (3.65%)	Other localized abdominal pain (3.48%)	Cough (3.35%)	Cough (3.33%)	AURTI (3.83%)
	Vertigo/dizziness (3.54%)	Vertigo/dizziness (3.54%)	Vertigo/dizziness (3.34%)	Cough (3.43%)	Gastric functional disorder (3.16%)	Physical examination (2.66%)	Non-insulin-dependent diabetes mellitus (3.33%)
	Other localized abdominal pain (3.40%)	Throat symptom/complaint (2.90%)	Throat symptom/complaint (2.90%)	Throat symptom/complaint (2.86%)	Vertigo/dizziness (2.98%)	Hypertension without complications (2.43%)	Anxiety (2.85%)
	Muscle pain (2.35%)	Cough (2.43%)	Gastric functional disorder (2.38%)	Gastric functional disorder (2.45%)	Other localized abdominal pain (2.52%)	Gastric functional disorder (2.41%)	Vertigo/dizziness (2.39%)
	Allergic rhinitis (2.23%)	Gastric functional disorder (2.32%)	Vertigo/dizziness (2.27%)	Abnormal inspection results NOS (2.20%)	Abnormal inspection results NOS (2.44%)	Vertigo/dizziness (2.37%)	Hypertension with complications (2.13%)
	Gastric functional disorder (2.20%)	Chest symptoms/complaint (2.01%)	Chest symptoms/complaint (1.99%)	Chest pain (2.00%)	Hypertension without complications (2.21%)	Health consultation (2.06%)	Physical examination (2.09%)
	Abdominal pain (1.93%)	Chest pain (1.96%)	Chest pain (1.90%)	Chest symptoms/complaint (1.93%)	Physical examination (2.01%)	Other localized abdominal pain (1.86%)	Sleep disorders (2.09%)
	Chest pain (1.92%)	Low back pain (1.71%)	Abnormal inspection results NOS (1.76%)	Low back pain (1.91%)	Throat symptom/complaint (1.83%)	Viral hepatitis (1.80%)	Dyslipidemia (2.08%)

Table 6 shows the proportion of psychological problems. Sleep disorder (P06) was the most common problem that ranked first in the groups over 20 years old. Anxiety (P74) and feeling anxious (P01) were also common, which altogether accounted for 30.02% in adolescents, 41.02% in young adults, 37.13% in adults, and 25.61% in the elderly. Depression (P76) and feeling depressed (P03) were less common, which accounted for 22.26% in young adults, 11.55% in adults, and 9.35% in the elderly, but it ranked first (33.17%) in the adolescent group.

**Table 6:** Top five psychological problems in each age group.

	**Pediatric (0–9)**	**Adolescent (10–19)**	**Young adult (20–44)**	**Adult (45–64)**	**Elderly (≥65)**
% of psychological problems in each age group	Symptoms/complaints of child behavior (33.33%)	Feeling depressed (24.21%)	Sleep disorder (26.89%)	Sleep disorder (40.43%)	Sleep disorder (45.72%)
	Feeling anxious/nervous (22.22%)	Sleep disorder (17.80%)	Anxiety (21.00%)	Anxiety (21.96%)	Anxiety (16.34%)
	Sleep disorder (22.22%)	Anxiety (16.34%)	Feeling anxious/nervous (20.02%)	Feeling anxious/nervous (15.17%)	Feeling anxious/nervous (9.27%)
	Anxiety (11.11%)	Feeling anxious/nervous (13.68%)	Feeling depressed (16.76%)	Feeling depressed (8.91%)	Feeling depressed (7.43%)
	Other psychological symptoms/complaints (11.11%)	Depression (8.96%)	Depression (5.50%)	Somatization disorder (6.88%)	Somatization disorder (7.17%)

## Discussion

The results revealed that general practitioners (GPs) in general outpatients' clinics served patients from all age groups, and health problems were distributed in all body systems. As mentioned previously, young adults accounted for the most significant proportion (60.02%) in all patients, and the average age of the patients in this study was 41 years 6 months, which is consistent with the census data in Shenzhen (4). In the University of Hong Kong-Shen Zhen Hospital, children's health problems are primarily dealt with by pediatricians rather than general practitioners. Thus, only a tiny percentage (0.09%) of the pediatric group seek medical advice at general outpatients' clinics.

Results from this study showed a distribution of health problems in body systems, and the top five chapters were “Respiratory,” “Digestive,” “Musculoskeletal,” “General,” and “Cardiovascular.” The result is similar to the study conducted in rural areas of Beijing, China (12). However, lower proportion of cardiovascular and respiratory problems and higher proportion of digestive problems were revealed in this study compared to those in rural areas of Beijing. The difference may be due to the diversity of gender and age distribution between the two studies. In Beijing, the proportion of older patients and cardiovascular problems was higher, and advanced age is well-known as a cardiovascular risk factor (14, 15).

The top 30 health problems accounted for over 50% of the total patients encountered, some of which were symptom descriptions and others were diagnostic descriptions. The majority of patients presented at primary care suffered from non-urgent health problems. However, some symptoms, such as chest pain, chest tightness, and vertigo, may be associated with life-threatening diseases, which accounted for about 4% of all the health problems. GPs are often patients' first contact and capable of dealing with latent diseases. In a previous study, GPs can effectively identify myocardial infarction patients with chest symptoms using HEART score, which have 91.8% sensitivity and 52.5% specificity (16). Timely identification and treatment are helpful to reduce mortality and improve the prognosis of myocardial infarction (17). Hence GPs should always remain alert for the potential critical diseases in primary care and be familiar with the process of diagnosis and treatment.

Acute upper respiratory infection (AURTI) was the most common diagnosis. At the same time, the cough was the most common complaint, which accounted for 9.80% of the health problems in all age groups, especially among patients under the age of 44. This result is a similar trend to previous studies in Hong Kong (18). Respiratory symptoms/diseases were usually the main reason for patients to visit in primary care (19), which deserved more attention on policy formulation and GP cultivation.

Although the average age of patients in Shenzhen city in this study is lower than in other regions of China (20, 21), chronic diseases, especially hypertension and diabetes, were still common, accounting for 12.21% in the adult group and 20.55% in the elderly group, respectively. Patients suffering from these chronic diseases can develop complications, especially cardiovascular diseases in the elderly. A previous study showed that rapid urbanization and economic development in China changed morbidity patterns, which led to poorer control of cardiovascular risk factors (22). The high prevalence of chronic diseases and adverse health outcomes related to them have raised GPs' awareness to intervene early, including lifestyle modifying, complications detection, continual monitoring, and support service provision.

In the distribution of cases by years, respiratory system diseases especially acute upper respiratory infection (AURTI) was the most common problem. But a rapid decline of AURI was found in 2020, as well as the total number of cases. It's may due to the emergence of COVID-19, which affected the regular clinical workflow in medical institutions (23). China managed to control its COVID-19 outbreak within several months, and the prevention and control measures require the patients with respiratory symptoms to visit the fever clinic instead of general outpatients' clinics (24, 25). As a result, the number of respiratory diseases has decreased, and chroinc diseases accounted for the main health problems in general outpatients' clinics after 2019.

The proportion of consultations for anxiety and depression in our study was 0.95 and 0.21%, respectively. Depression accounts for a higher proportion in adolescents, and anxiety is higher in young adults and adults. These figures seem low in our study compared with the high prevalence (9.3%) of mental health problems reported in China (26). These results may be due to the poor psychological knowledge and training of GPs, which increase the rate of misdiagnosis of mental health problems (27). To solve the problem, the World Association of Family Doctors issued a declaration calling for the integration of mental health services in primary care (28). The declaration recommends training GPs to help them better identify and respond to patients with mental disorders in primary care, reducing stigma for mental health patients and having a better follow-up on mental disorders.

The results above are significant as they reflect the most frequent health problems in the primary health services, which will objectively guide general practitioners to pay more attention to the commonest problems bothering most residents.

### Limitation

It is essential to recognize the limitations of this study. Firstly, the consultation dates were insufficient in the study due to the half-baked medical information system, especially the interventions, consultation length and expenses, which were helpful to analyze the treatment process of patients further. Secondly, as some patients' data were incomplete leading to possible selection bias. Finally, the study is based on one hospital and may not be generalizable to the whole Shen Zhen community. However, we did this because it was convenient to get the data from this hospital and we hope that with large sample size that it can shed some important information on the overall Shen Zhen community. The limitations above may influence the estimation of symptom prevalence and disease frequency.

### Conclusions

This study has presented the diversity of health problems at the general outpatients' clinic of the University of Hong Kong-Shenzhen Hospital, which reflected the comprehensive care provided by GPs. The data will contribute to understanding the demands of patients and the work contents of GPs in Shenzhen city to provide more reference for the medical reform of primary care.

## Data availability statement

The original contributions presented in the study are included in the article/supplementary material, further inquiries can be directed to the corresponding author.

## Author contributions

KC and ZL designed the study and wrote up the manuscript. WY and RL were responsible for supervising the whole project and compiling the manuscript. YZ and ZC assisted in the design of study and polished the language. NP assisted in polishing and typesetting of manuscript. All authors had read and approved the manuscript.

## Conflict of interest

The authors declare that the research was conducted in the absence of any commercial or financial relationships that could be construed as a potential conflict of interest.

## Publisher's note

All claims expressed in this article are solely those of the authors and do not necessarily represent those of their affiliated organizations, or those of the publisher, the editors and the reviewers. Any product that may be evaluated in this article, or claim that may be made by its manufacturer, is not guaranteed or endorsed by the publisher.

## References

[1] Li X Lu J Hu S Cheng KK De Maeseneer J Meng Q. The primary health-care system in China. Lancet. (2017) 390:2584–94. 10.1016/S0140-6736(17)33109-4 29231837

[2] Yip W Fu H Chen AT Zhai T Jian W Xu R. 10 years of health-care reform in China: progress and gaps in Universal Health Coverage. Lancet. (2019) 394:1192–204. 10.1016/S0140-6736(19)32136-1 31571602

[3] Yan C Liao H Ma Y Wang J. The impact of health care reform since 2009 on the efficiency of primary health services: a provincial panel data study in China. Front Public Health. (2021) 9:735654. 10.3389/fpubh.2021.735654 34746081 PMC8569255

[4] Census Rot7P. Shenzhen Statistics Bureau, Shenzhen (2020).

[5] Liang C Mei J Liang Y Hu R Li L Kuang L. The effects of gatekeeping on the quality of primary care in Guangdong Province, China: a cross-sectional study using primary care assessment tool-adult edition. BMC Fam Pract. (2019) 20:93. 10.1186/s12875-019-0982-z 31272392 PMC6610915

[6] Li X Krumholz HM Yip W Cheng KK De Maeseneer J Meng Q. Quality of primary health care in China: challenges and recommendations. Lancet. (2020) 395:1802–12. 10.1016/S0140-6736(20)30122-7 32505251 PMC7272159

[7] Xu DR Hu M He W Liao J Cai Y Sylvia S. Assessing the quality of primary healthcare in seven Chinese provinces with unannounced standardised patients: protocol of a cross-sectional survey. BMJ Open. (2019) 9:e023997. 10.1136/bmjopen-2018-023997 30765399 PMC6398795

[8] de Luca K Hogg-Johnson S Funabashi M Mior S French SD. The profile of older adults seeking chiropractic care: a secondary analysis. BMC Geriatr. (2021) 21:271. 10.1186/s12877-021-02218-6 33892643 PMC8066480

[9] Savran O Godtfredsen NS Sørensen T Jensen C Ulrik CS. Comparison of characteristics between ICS-treated COPD patients and ICS-treated COPD patients with concomitant asthma: a study in primary care. Int J Chron Obstruct Pulmon Dis. (2020) 15:931–7. 10.2147/COPD.S241561 32425518 PMC7196437

[10] Gusso G. The International Classification of Primary Care: capturing and sorting clinical information. Cien Saude Colet. (2020) 25:1241–50. 10.1590/1413-81232020254.30922019 32267427

[11] Wan E Chin WY Yu E Chen J Tse ET Wong CK. Retrospective cohort study to investigate the 10-year trajectories of disease patterns in patients with hypertension and/or diabetes mellitus on subsequent cardiovascular outcomes and health service utilisation: a study protocol. BMJ Open. (2021) 11:e038775. 10.1136/bmjopen-2020-038775 33550225 PMC7925871

[12] Liu Y Chen C Jin G Zhao Y Chen L Du J. Reasons for encounter and health problems managed by general practitioners in the rural areas of Beijing, China: a cross-sectional study. PLoS ONE. (2017) 12:e0190036. 10.1371/journal.pone.0190036 29267362 PMC5739459

[13] Xiaoping XU Deng D Dongge KE Lin L Qianhui YU Yonghong GU. Research on patient safety culture in the university of hong kong, shenzhen hospital. Chinese Hospitals.34631518

[14] Grilo GA Shaver PR Stoffel HJ Morrow CA Johnson OT Iyer RP. Age- and sex-dependent differences in extracellular matrix metabolism associate with cardiac functional and structural changes. J Mol Cell Cardiol. (2020) 139:62–74. 10.1016/j.yjmcc.2020.01.005 31978395 PMC11017332

[15] Park JH Seo MW Jung HC Song JK Lee JM. Association between health-related physical fitness and respiratory diseases in adolescents: an age- and gender-matched study. Int J Environ Res Public Health. (2021) 18. 10.3390/ijerph18126655 34205703 PMC8296502

[16] Johannessen TR Atar D Vallersnes OM Larstorp A Mdala I Halvorsen S. Comparison of a single high-sensitivity cardiac troponin T measurement with the HEART score for rapid rule-out of acute myocardial infarction in a primary care emergency setting: a cohort study. BMJ Open. (2021) 11:e046024. 10.1136/bmjopen-2020-046024 33627355 PMC7908281

[17] Reed GW Rossi JE Cannon CP. Acute myocardial infarction. Lancet. (2017) 389:197–210. 10.1016/S0140-6736(16)30677-8 27502078

[18] Lo YY Lam CL Mercer SW Fong DYT Lee A Lam TP. Patient morbidity and management patterns of community-based primaryhealth care services in Hong Kong. Hong Kong Med J. (2011) 17(3 Suppl 3):33–7. 21673358

[19] Huibers LA Moth G Bondevik GT Kersnik J Huber CA Christensen MB. Diagnostic scope in out-of-hours primary care services in eight European countries: an observational study. BMC Fam Pract. (2011) 12:30. 10.1186/1471-2296-12-30 21569483 PMC3114765

[20] Dawes M. Symptoms, reasons for encounter and diagnoses. Family practice is an international discipline. Fam Pract. (2012) 29:243–4. 10.1093/fampra/cms018 22421059

[21] Ding H Chen Y Yu M Zhong J Hu R Chen X. The effects of chronic disease management in primary health care: evidence from rural China. J Health Econ. (2021) 80:102539. 10.1016/j.jhealeco.2021.102539 34740053

[22] Du X Patel A Anderson CS Dong J Ma C. Epidemiology of cardiovascular disease in China and opportunities for improvement: JACC International. J Am Coll Cardiol. (2019) 73:3135–47. 10.1016/j.jacc.2019.04.036 31221263

[23] Xu Z Ye Y Wang Y Qian Y Pan J Lu Y. Primary care practitioners' barriers to and experience of COVID-19 epidemic control in china: a qualitative study. J Gen Intern Med. (2020) 35:3278–84. 10.1007/s11606-020-06107-3 32869200 PMC7458355

[24] Gao J Zhang P. China's public health policies in response to COVID-19: from an “authoritarian” perspective. Front Public Health. (2021) 9:756677. 10.3389/fpubh.2021.756677 34976920 PMC8714736

[25] Li D. Challenges and responsibilities of family doctors in the new global coronavirus outbreak. Fam Med Commun Health. (2020) 8:e000333. 10.1136/fmch-2020-000333 32148740 PMC7046369

[26] Huang Y Wang Y Wang H Liu Z Yu X Yan J. Prevalence of mental disorders in China: a cross-sectional epidemiological study. Lancet Psychiatry. (2019) 6:211–24. 10.1016/S2215-0366(18)30511-X 30792114

[27] Liang D Mays VM Hwang WC. Integrated mental health services in China: challenges and planning for the future. Health Policy Plan. (2018) 33:107–22. 10.1093/heapol/czx137 29040516 PMC5886187

[28] Greenhalgh T. WHO/WONCA report - integrating mental health in primary care: a global perspective. London J Prim Care. (2009) 2:81–2. 10.1080/17571472.2009.11493254 26042178 PMC4453694

